# On the Use of Iodide of Potassium in the Treatment of Luchorrhœa by Injections

**Published:** 1858-06

**Authors:** 


**On the use of Iodide of Potassium in the Treatment of Leucorrhoea by Injections.**

The wise man said, "There is nothing new under the sun," and the saying applied to the practice of Medicine in the present age, can admit of no refutation, and indeed of but little opposition.

Most of the remedies or curative agents lately brought into notice, more especially those which are published to the world as new discoveries, may be proved to have been used in very remote periods; and many too have been well known among the teachers of medicine, in the very infancy of the art. The present age, perhaps, more than any other, has been characterized by the successful diligence and zeal which it has displayed in researches. The efforts made in the various arts and sciences, have been often rewarded by great and important discoveries, and none of the branches of knowledge can boast of more successful improvements than those which have been connected with medical inquiries. While concession to the truth of this opinion must be very generally acknowledged, it will also be allowed, the assiduity and ardor of pursuit after novelties, especially in the healing art, has been almost everywhere so exclusively directed to recent or modern innovations in practical as well as theoretical principles, that much very important though ancient medical skill, communicated by the writings of an early age, has been disregarded, or ignorantly confounded with pretended new discoveries; the new medical author or practitioner claiming the merit and honor, of first introducing as a beneficial novelty, some plan or method well known to his predecessors, and recorded in works yet extant, and of easy attainment. It is certain that many important articles in the materia medica, apparently of opposite tendency to one another, are judiciously used in morbid affections. To account satisfactorily for this fact, theory alone will not suffice, but experience will, in such instances, guide the physician's conduct, without regard to any theory; and the beneficial result of the use of seemingly opposite medicines, will often do away the extravagant respect so uniformly shown to theoretical reasoning in the practice of young physicians.

But my present object being to awaken the attention of my medical brethren to an article which I deem of greater importance and efficacy, and possessed of more valuable properties than are generally ascribed to it. This remedy is the iodide of potassium.

I will now give the history of three cases which came under my care: although they may be imperfect in many particulars, yet I hope they are of sufficient minuteness and correctness to occupy the sphere they are intended for, in the mind's eye of the reader.

In the spring of 1856, Jane, a negress of Mr. E. S. Miller, *œt.* 30 years, of a leucophlegmatic temperament, came under my treatment. Two years ago she was attacked with dysmenorrhœa, followed soon after by prolapsus uteri, with a leucorrhœal discharge. When she came under my care the leucorrhœal discharge was of more than an ordinary quantity usually met with in such cases. When the discharge came in contact with the external parts, it produced excoriation. Previous to the appearance of the catamenia, the uterus would sink low into the pelvis, frequently making its exit beyond the labia externia. The catamenia was of the usual character and quantity for the first two or three days; then it would change its form in regard to quantity, and assume that of menorrhagia, never yielding but to the action of remedies. Such is the general history of the above case when it came under my care.

My first object being to relieve the dysmenorrhœa and prolapsus of the uterus, which I accomplished after "so long a a time," but with no diminution of the leucorrhœal discharge. I followed the usual course of treatment for its relief, but with no avail. Relying solely on an alterative course of treatment for its cure, my mind naturally led to the use of iodide of potassium. I reasoned with myself thus: If the iodide of potassium is such an efficient alterative when taken by the mouth and addressed to the constitution generally, would its action not be more potent, if brought within direct contact with the diseased parts? Acting upon these premises, I commenced the use of iodide of potassium by injections per vaginam; and much to my surprise (though of an agreeable character), the discharge made a speedy surrender. It has not made its re-appearance, now nearly a year since.

1857. Mrs. S., *æt.* about 21 years, of a sanguine temperament, has suffered much since her last confinement (six months ago) with leucorrhœa, of an ordinary character. Ordered a solution of iodide of potassium by injections three or four times a day. The discharge was soon relieved.

Mrs. C., *æt.* about 28 years; leucophlegmatic temperament. Confined about seven weeks since with her fourth child. Suffered much after her accouchment with ovaritis. Leucorrhœal discharge somewhat profuse, and of a thin, white appearance. Ordered iodide of potassium by injections. Discharge checked on the fourth or fifth day.

The quantity of iodide of potassium was as follows: dr. iss to a half pint of aqua puræ.

